# Design of Lattice-Matched InAs_1−*x*_Sb*_x_*/Al_1−*y*_In*_y_*Sb Type-I Quantum Wells with Tunable Near-To Mid-Infrared Emission (2–5 μm): A Strain-Optimized Approach for Optoelectronic Applications

**DOI:** 10.3390/nano16020147

**Published:** 2026-01-22

**Authors:** Gerardo Villa-Martínez, Julio Gregorio Mendoza-Álvarez

**Affiliations:** 1 Sección de Estudios de Posgrado e Investigación, ESIME-Ticomán, Instituto Politécnico Nacional, Ciudad de México 07340, Mexico; 2 Departamento de Física, Centro de Investigación y de Estudios Avanzados del IPN, Ciudad de México 07360, Mexico; julio.mendoza@cinvestav.mx

**Keywords:** InAsSb, AlInSb, quantum wells, near-infrared, type-I, band offset

## Abstract

We propose a strain-optimized design strategy for lattice-matched InAs_1−*x*_Sb*_x_*/Al_1−*y*_In*_y_*Sb Type-I quantum wells (QWs) that emit across the near-to mid-infrared spectrum (2–5 µm). By combining elastic strain energy minimization with band offset calculations, we identify Type-I alignment for Sb contents (*x* ≤ 0.40) and In contents (0.10 < *y* ≤ 1). At the same time, Type-II dominates at higher Sb compositions (*x* ≥ 0.50). Using the transfer matrix method under the effective mass approximation, we demonstrate precise emission tuning via QW thickness (*L*_W_) and compositional control, achieving a wavelength coverage of 2–5 µm with <5% strain-induced energy deviation. Our results provide a roadmap for high-efficiency infrared optoelectronic devices, addressing applications in sensing and communications technologies.

## 1. Introduction

**Figure 1:**
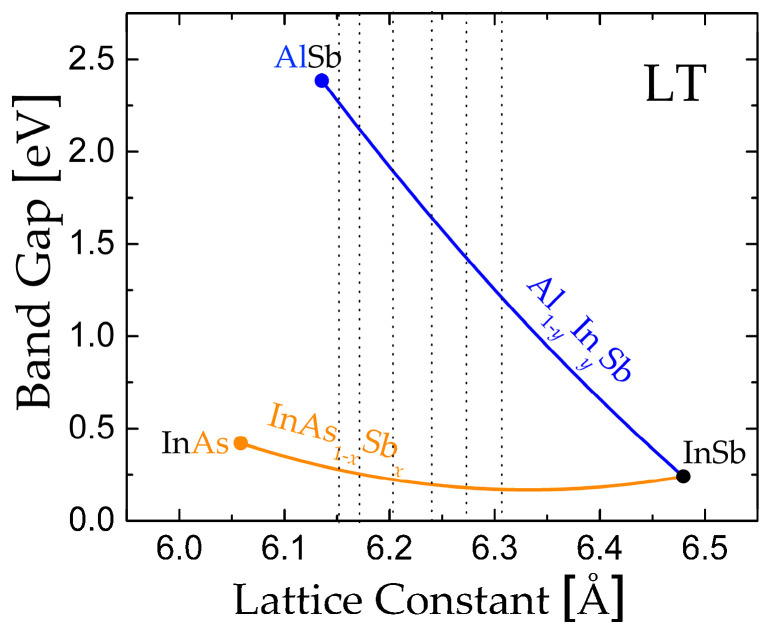
The band gap energy as a function of the lattice constant for InAs_1−*x*_Sb_*x*_ and Al_1−*y*_In*_y_*Sb. The vertical line marks lattice-matched compositions.

Antimonide-based semiconductors have emerged as a cornerstone for infrared optoelectronics due to their tailorable band gaps and high carrier mobility. Among these, InAsSb alloys are particularly promising for the 2–5 µm range, bridging the near- and mid-infrared spectral windows critical for molecular sensing and free-space communication [1]. InSb-based QWs exhibit record electron mobilities exceeding 200,000 cm^2^/Vs at low temperatures (LT) and room temperature (RT) mobilities of 78,000 cm^2^/Vs [2,3], attributed to their ultra-low effective mass (m* = 0.013*m*_e_) and high Fermi velocities [4]. However, the incorporation of As (e.g., InAs_0.10_Sb_0.90_) shifts alignment to Type II [5], reducing resistivity but impeding light emission efficiency. Recent studies demonstrate that InAsSb/AlInSb heterostructures grown by molecular beam epitaxy (MBE) achieve exceptional interfacial and strong carrier confinement [1,6], yet thermal instability and lattice-mismatch induced defects persist, especially on GaAs substrates [7]. Critical to performance is strain management at the InAsSb/AlInSb interface, where mismatched lattice parameters and thermodynamic differences degrade QW integrity [8,9]. The InAs_1−*x*_Sb*_x_* ternary alloy exhibits a pronounced bandgap (*E*_g_) bowing effect, enabling tunable emission across the 2.95–5.17 µm range [2], but optimal compositions (e.g., *x* ≈ 0.2) demand lattice matching (*y* = 1.2245*x* − 0.2245, Figure 1) to minimize strain. While InAs_1−*x*_Sb*_x_* QWs (*x* = 0.4–0.6) offer improved thermal stability [10], achieving Type-I alignment requires precise control of composition and QW thickness (*L*_w_) to balance band offsets and dislocation suppression [1,11].

Despite progress, designing strain-optimized, Type-I InAsSb/AlInSb QWs with tunable emission (2–5 µm) remains unresolved, particularly for high-efficiency light-emitting devices. Here, we combine elastic strain energy minimization with band offset calculations to predict Type-I alignment and suppress Type-II transitions. Using transfer matrix methods, we demonstrate broadband emission tuning via *L*_w_ and composition for IR optoelectronic applications.

## 2. Calculation Procedures

Research shows that In-As-Sb alloys have a shared cation group (In) formed by substituting group V atoms during crystal growth. Similarly, Al-In-Sb alloys share an anion group (Sb) formed by substituting group III atoms [8]. Thus, InAs_1−*x*_Sb*_x_* and Al_1−*y*_In*_y_*Sb alloys are combinations of compounds (InAs, InSb, AlSb) [12]. All physical parameters were computed utilizing Virtual Crystal Approximation (VCA). In this context, a VCA crystal is regarded as possessing an average effective crystal potential, which is determined based on a lattice constant as defined by Vegard’s Law. Generally, the properties of a ternary alloy can be linearly interpolated between those of its binary constituents, indicating that the chemical bonding of atoms within the alloy transitions smoothly between the values of the endpoint compounds. This assumption serves as the foundation of the VCA. The VCA offers an efficient framework for predicting alloy properties. It models the alloy with a perfectly periodic average potential, ignoring short-range disorder or local composition fluctuations common in real ternary alloys. For parameters with nonlinear composition dependence, linear interpolation (Equation (1)), even with empirical bowing parameters, can introduce inaccuracies in properties such as deformation potentials or effective masses, especially at intermediate compositions. The main errors stem from oversimplified band edges, possibly leading to underestimates of band-tailing and transition energies. Despite this, VCA effectively identifies trends, phase boundaries, and design principles, as shown in this work [13]. Accordingly, we employed the following formulas:

(1) $$P_{InAs1-xSbx}=\left(1\;-x\right)P_{InAs}+xP_{InSb},\;P_{Al1-yInySb}=\left(1\;-y\right)P_{AlSb}+yP_{InSb}$$

where $P_{InAs}$, $P_{InSb}$, and $P_{AlSb}$ are parameters associated with binary compounds that form a given ternary alloy, for example, the lattice constant, effective mass, elastic stiffness, band gap energy (see Table 1); in the case of *E*_g_ of ternary alloys, as a function of composition, the non-linear deviation occurring during alloy formation is accounted for by including a bowing parameter specific to each ternary alloy. The bowing parameters for the alloys, *b*_InAsSb_ = 0.60 eV and *b*_AlInSb_ = 0.43 eV, are well-known values from the literature and are consistent with Vurgaftman et al. [8], who suggest that *b*_InAsSb_ = 0.67 eV and *b*_AlInSb_ = 0.43 eV. The minor difference in InAsSb, within the typical 0.6–0.7 eV range, does not impact the trends. Using these standard parameters ensures our bandgap calculations align with recognized data for III-V semiconductors.

## 3. Results and Discussion

### 3.1. Elastic Strain Energy in InAs_1−x_Sb_x_/Al_1−y_In_y_Sb Heterostructures

Lattice matching is critical for minimizing defects in InAs_1−*x*_Sb*_x_*/Al_1−*y*_In*_y_*Sb quantum wells (QWs). The in-plane strain ($\varepsilon_{∥}$) at the interface is governed by

(2) $$\varepsilon_{∥}=\varepsilon_{yy}=\varepsilon_{zz}=\frac{a_{AlInSb}(y)-a_{InAsSb}(x)}{a_{AlInSb}(y)}$$

where *a* is the lattice constant (Table 1). For (100)-oriented growth, $\varepsilon_{∥}$ induces tetragonal distortion, with out-of-plane strain ($\varepsilon_{⊥}$) given by

(3) $$\varepsilon_{⊥}=\varepsilon_{xx}=-2\frac{C_{12}}{C_{11}}\varepsilon_{∥}$$

Strain energy minimization is achieved via calculations of the elastic potential energy given by the following [15]:

(4) $$\begin{array}{lll} E_{strain} & =E\left(x,y\right) & =\frac{1}{2}C_{11}\left(\varepsilon_{xx}^{2}+\varepsilon_{yy}^{2}+\varepsilon_{zz}^{2}\right)+C_{12}\left(\varepsilon_{xx}\varepsilon_{yy}+\varepsilon_{zz}\varepsilon_{yy}+\varepsilon_{xx}\varepsilon_{zz}\right)\\ & & +\frac{1}{2}C_{44}\left(\varepsilon_{xy}^{2}+\varepsilon_{yz}^{2}+\varepsilon_{xz}^{2}\right) \end{array}$$

The elastic stiffness coefficients ($C_{11}$, $C_{12}$, $C_{44}$) of InAs_1−*x*_Sb*_x_* and Al_1−*y*_In*_y_*Sb are expressed as $C_{InAsSb}=\left(1-x\right)C_{InAs}+xC_{InSb}$ and $C_{AlInSb}=\left(1-y\right)C_{AlSb}+yC_{InSb}$. The numerical values of the stiffness coefficients used in the calculations are presented in Table 1. For the (100)-oriented heterostructure, the InAs_1−*x*_Sb*_x_* epilayer undergoes biaxial in-plane strain, inducing tetragonal distortion. Additionally, we assumed that the Al_1−*y*_In*_y_*Sb epilayer is infinitely thick, thereby eliminating any in-plane shear strain on the Al_1−*y*_In*_y_*Sb. The strain tensor was computed using the reference [16], incorporating both normal and shear components, resulting in a (100)-oriented heterostructure, $\varepsilon_{yz}=\varepsilon_{yx}=\varepsilon_{xz}=0$.

In the absence of lattice matching, residual strain significantly impacts both the electronic structure and the luminescence efficiency of the QWs. Electronically, strain modifies the band gap energy via the deformation potential and induces splitting of the valence bands into heavy-hole (HH) and light-hole (LH) subbands [8]. This alters the density of states, shifts transition energies, and can change the effective mass of carriers. In extreme cases, strain can even modify the band alignment, potentially driving a Type-I heterostructure into a Type-II configuration, thereby spatially separating electrons and holes and reducing the probability of radiative recombination [5]. High strain energy promotes the generation of misfit dislocations at the heterointerface. These defects act as non-radiative recombination centers, drastically quenching photoluminescence intensity and degrading internal quantum efficiency [17]. Furthermore, accumulated strain may lead to partial or complete relaxation of the quantum well layer, resulting in interfacial roughness and increased carrier scattering [18]. Therefore, minimizing strain through lattice matching is not merely a structural concern but is essential for achieving high radiative efficiency and predictable emission characteristics in InAsSb/AlInSb QW-based devices.

While this work focuses on the ideal lattice-matched condition for maximal performance, practical device integration on conventional substrates, or for specific bandgap targets, may require operating under some residual strain. In such cases, advanced strain compensation strategies can be employed to mitigate defect formation. These include the use of strain-balanced superlattices, in which alternating tensile and compressive layers achieve a net zero average strain on the substrate, and graded buffer layers that gradually transition the lattice constant from the substrate to the active region, thereby filtering threading dislocations [19,20]. The design principles established here for individual QWs can be extended within these more complex, strain-engineered architectures, offering a pathway to integrate tunable InAsSb/AlInSb emitters with a broader range of photonic platforms.

**Figure 2:**
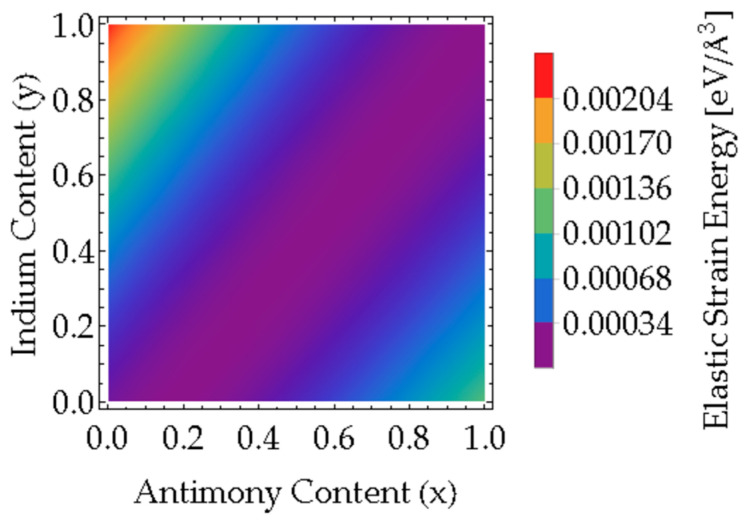
Elastic strain energies of the InAs_1−*x*_Sb*_x_*/AlIn*_y_*Sb_1−*y*_ interface were calculated for all combinations of the Sb(*x*) and In(*y*) contents.

Figure 2 maps $E_{strain}$ across all (*x*, *y*) compositions, revealing a minimum (purple region) at the lattice-matched condition y=1.2245x−0.2245. Meanwhile, the maximum strain energy (red region) is observed for InAs (x=0) on InSb (y=1), where the considerable tensile strain arises from the mismatch between InAs (smaller lattice parameter) and InSb (most significant lattice parameter). Based on reports from the literature for high-quality, defect-free III–V quantum wells, the acceptable in-plane strain is typically below 1–2%, corresponding to an elastic strain energy of less than 1–2 meV/Å^3^ in Figure 2. Compositions lying within the dark blue/purple regions of Figure 2 satisfy this criterion and are thus considered experimentally feasible for device implementation.

### 3.2. Band Alignment and Type-I/II Criteria in InAs_1−x_Sb_x_/Al_1−y_In_y_Sb Interface

**Figure 3:**
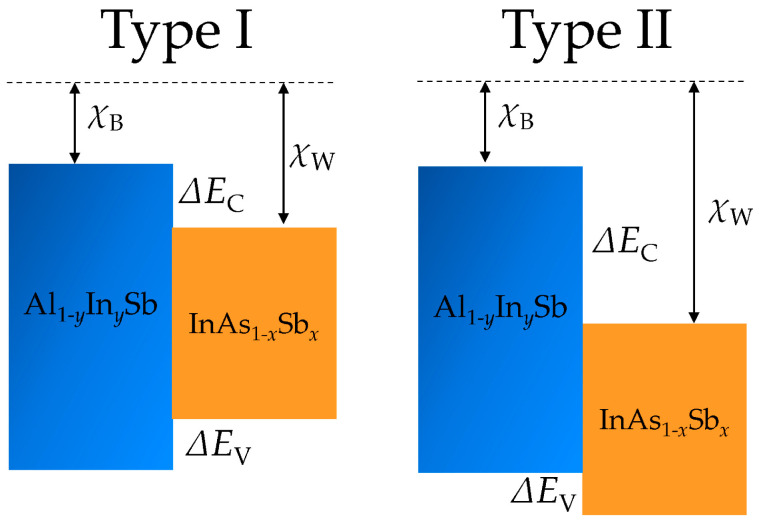
The band alignments for Al_1−*y*_In*_y_*Sb/InAs_1−*x*_Sb*_x_* heterojunctions can be Type I or Type II.

The heterostructure type can be identified using the electron affinity rule, where the offset in the conduction band at the interface equals the difference in the electron affinity values of the two compounds, InAs_1−*x*_Sb*_x_*/Al_1−*y*_In*_y_*Sb. Band offsets refer to the relative alignment of the energy bands at the interface of a heterojunction—a junction formed between two distinct semiconductor materials. This phenomenon is crucial in optoelectronic device design, as it governs the behavior of electrons and holes at the interface, directly influencing carrier recombination dynamics, light emission efficiency, and electrical conductivity. Band offsets are commonly determined using theoretical models, such as Anderson’s rule and linear theory, which account for intrinsic semiconductor properties and interfacial effects. Anderson’s rule estimates band alignment using fundamental material parameters, including electron affinity and bandgap values, providing a first-order approximation of the band alignment, band offsets [21]. Meanwhile, linear theory refines this approach by incorporating electronic structure details and bonding characteristics at the interface, enabling more accurate predictions for complex heterostructures [22]. Their continued relevance stems from their adaptability to novel material systems and their utility in guiding the design of interfaces for advanced optoelectronic applications. Heterojunctions exhibit two primary band alignment configurations (see Figure 3): Type I (straddling) and Type II (staggered), depending on the alignment of the conduction and valence bands [23]. In Type I, the conduction band minimum (CBM) and valence band maximum (VBM) of one material are fully nested within the bandgap of the other. This configuration localizes both electrons and holes in the same material, enhancing radiative recombination. In Type II, the CBM of one material aligns within the band gap of the other, creating a spatial separation of electrons and holes. This promotes charge transfer across the interface, which is suitable for photovoltaic and photocatalytic devices.

By using the electron affinity rule [21], in which the offset in the conduction band (ΔE_C_) at the interface is equal to the difference in electron affinity values of the two semiconductors, in our case, for InAs_1−*x*_Sb*_x_* and AlIn_1−*y*_Sb*_y_*, defined as

(5) $$∆E_{C}=\chi_{W}-\chi_{B}={\chi_{InAsSb}-\chi }_{AlInSb}$$

While Anderson’s rule provides a reliable first-order estimate based on electron affinity differences, it does not account for interface dipole effects, strain-induced band edge modifications, or chemical bonding disparities, which can be significant in polar, mismatched systems such as InAsSb/AlInSb. More advanced first-principles calculations or experimental measurements would be required for precise quantitative alignment, but are beyond the scope of this design-oriented study.

In addition, the following expression must be fulfilled, $∆E_{C}+∆E_{V}=E_{g,B}-E_{g,W}$, where $∆E_{V}$ corresponding to the offset in the valence band; $∆E_{C}+∆E_{V}=E_{g,AlInSb}-E_{g,InAsSb}$.

Following the linear approximation, we can express the band offsets as follows:

(6) $$∆E_{C}=∆E_{C}\left(x,y\right)={\chi_{InAsSb}-\chi }_{AlInSb}=\left(1-x\right)\chi_{InAs}+x\chi_{InSb}-\left(1-y\right)\chi_{AlSb}-\;y\chi_{InSb}$$

and

(7) $$∆E_{V}=∆E_{V}\left(x,y\right)=E_{g,AlInSb}\left(y\right)-E_{g,InAsSb}\left(x\right)-∆E_{C}\left(x,y\right)$$

**Figure 4:**
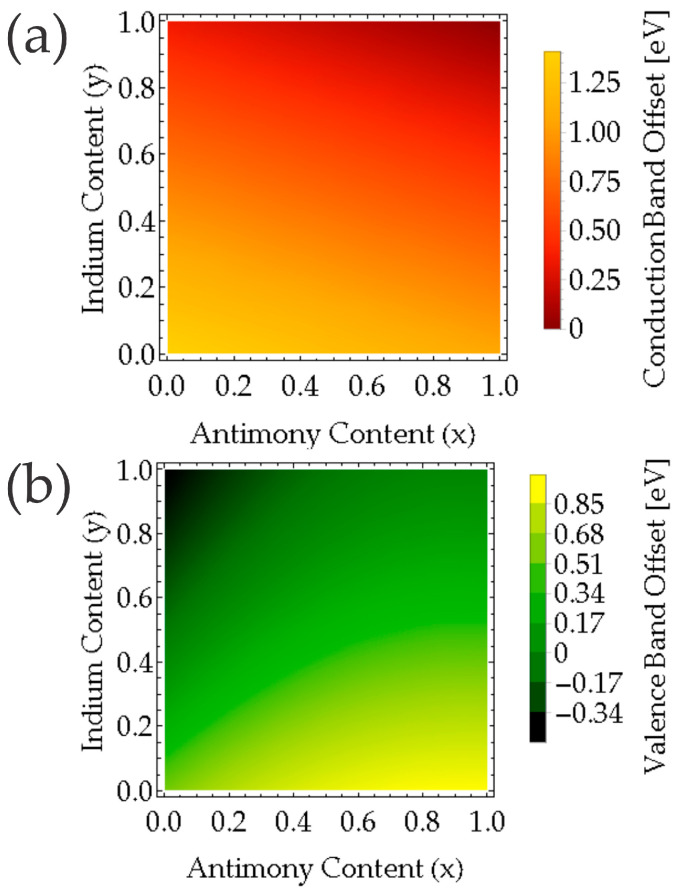
Evolution of the band alignment in evolution of the band alignment in InAs_1−*x*_Sb*_x_*/Al_1−*y*_In*_y_*Sb interface. (**a**) The conduction band offset, (**b**) the valence band offset.

The values employed for the calculation are presented in Table 1. Figure 4a displays the conduction band offset for InAs_1−*x*_Sb*_x_*/AlIn*_y_*Sb_1−*y*_ interface for all combinations of Sb (*x*) and In(*y*) contents. We can observe that $∆E_{C}>0$. Figure 4b shows the valence band offset for InAs_1−*x*_Sb*_x_*/AlIn*_y_*Sb_1−*y*_ interface for all combinations of Sb and In. InAs_1−*x*_Sb*_x_*/AlIn*_y_*Sb_1−*y*_ interface presents an $∆E_{V}<0$, indicating that for this region, the interface is Type II, and, also, $∆E_{V}>0$ corresponds to interface Type I.

It can be concluded that Type I is obtained for *y* ≤ 0.40 and 0 ≤ *x* ≤1; for *y* > 0.40, the antimony content (*x*) range can be found for 0.10 < *x* ≤ 1. While Type II is allowed for *y* ≥ 0.50, the antimony content (*x*) range can be found for 0.10 < *x* ≤ 1.

### 3.3. Tuning of Excitonic Transitions in Lattice-Matched Quantum Wells

**Figure 5:**
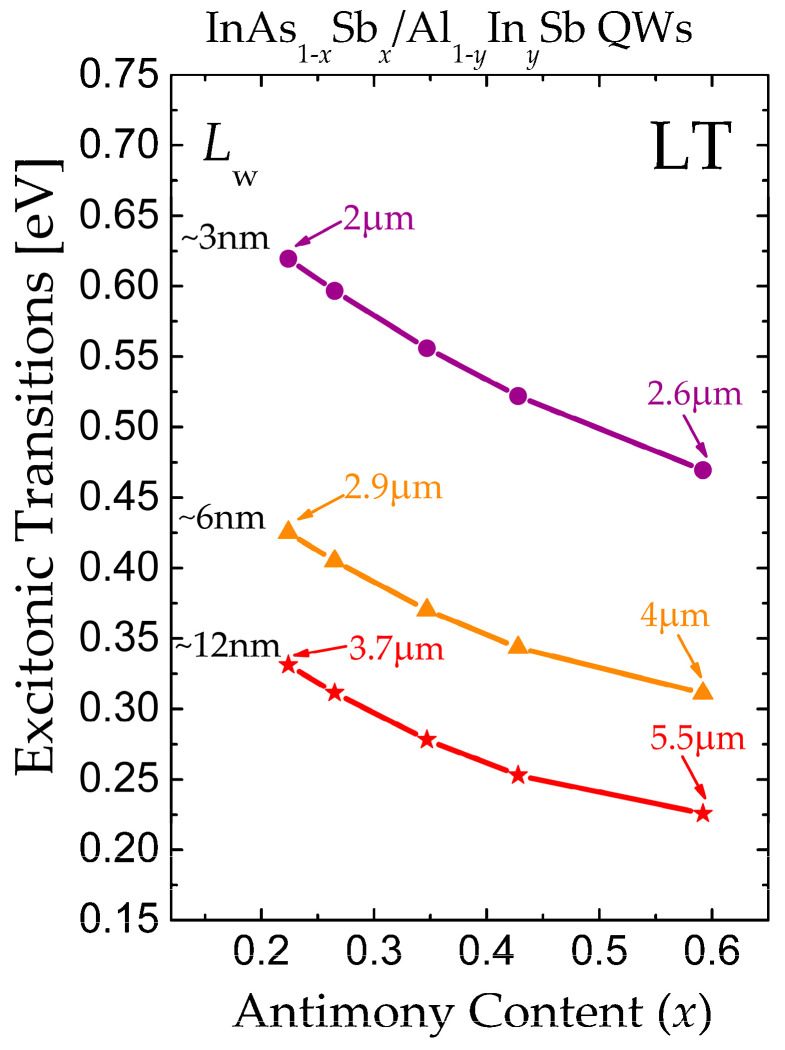
Calculated energies of the excitonic transitions under lattice match conditions for (*x*, *y*) compositions. The transition can be tuned by modifying the thickness of the QW (*L*w) and the content of (Sb, In). Five latticed-matched compositions are represented by solid line with purple circles (*L*_w_ = 3 nm), solid line with orange triangles (*L*_w_ = 6 nm) and solid line with red starts (*L*_w_ = 12 nm).

We have calculated the QW energy levels of QWs; we used the transfer matrix method under the effective mass envelope function approximation. The values used are shown in Table 1. For general calculations, we considered a QW with an InAs_1−*x*_Sb*_x_* central region and Al_1−*y*_In*_y_*Sb barriers under lattice-matched conditions, presenting a Type-I band alignment. The assumption of an infinitely thick, unstrained first (bottom) barrier is a standard simplification in envelope-function calculations, ensuring complete carrier confinement within the QW. In practice, barrier thicknesses exceeding 30 nm are typically sufficient to approximate this condition, with negligible coupling between adjacent wells or quantization effects in the barriers themselves. We have deemed excitonic transitions, and the excitonic binding energy was calculated following ref. [24]. The energy of the excitonic transition at low temperature can be expressed as *E*_exc_(LT) = *E*_g_(LT) + *E*_1e_ + *E*_1hh_ − *E*_1s_, where *E_g_*(LT) is the bandgap at low temperature of the QW material, *E*_1*e*_ and *E*_1*hh*_ are the ground states for electrons and heavy holes in their respective quantum wells, and *E*_1*s*_ is the exciton binding energy. The analytical model for exciton binding energy used in this work [24] provides a reliable estimate for strongly confined excitons in Type-I QWs, with an expected accuracy within 5 meV for the well width and composition range considered. This uncertainty does not affect the overall trends in tunability or the identification of Type-I/II transitions. In Figure 5, we observe the excitonic transition of InAs_1−*x*_Sb*_x_*/Al_1−*y*_In*_y_*Sb QW Type-I under lattice-matched conditions. As a reference, if we choose *x* = 0.224, then the lattice matching in content of the barrier will be *y* = 0.5 (lattice-matched Al_0.95_In_0.5_Sb/InAs_0.776_Sb_0.224_/Al_0.95_In_0.5_Sb QW Type I). From this figure, we can see that by choosing *y* = 0.5 in the QW, we can tune the emission to a range of 2–3.7 µm. Figure 5 reveals tunable excitonic transitions (2–5.5 µm) for five lattice-matched (*x*, *y*) compositions, (*x*, *y*) = (0.224, 0.05), (0.265, 0.10), (0.347, 0.20), (0.428, 0.30), and (0.592, 0.50), indicated by circles, triangles, and stars. For each (*x*, *y*) pair, we varied the thickness of QW (*L*_w_), from ~3 to ~12 nm. Notably, the (*x* = 0.224, *y* = 0.05) configuration achieves 3.7 µm emission at *L*_w_ = 12 nm, and Type-I alignment persists up to *x* = 0.40, beyond which Type-II dominates due to valence band crossover (Figure 4b), suggesting a trade-off between tunability and radiative efficiency. To ensure the experimental viability of the proposed designs, the strain energy analysis must be considered alongside the critical thickness for dislocation formation. Exceeding this thickness, as estimated by the Matthews–Blakeslee criterion for lattice-mismatched systems, generates misfit dislocations that severely degrade optical efficiency. The strain minimized compositions identified here inherently favor operation below the critical thickness, providing a practical pathway for growing defect-free, high-efficiency InAsSb/AlInSb quantum well structures [25,26]. From this figure, we can see that by choosing the adequate thickness of QW (*L*_w_) and In content, we can tune the emission from 2 to 5.5 µm.

The theoretical framework and predictions presented in this work are consistent with key experimental studies on InAsSb/AlInSb QW structures. Our identification of the transition from Type-I to Type-II alignment at higher Sb compositions (*x* ≥ 0.50) is strongly supported by the transport property analysis of Manago et al. [7,12]. Their work on InAs*_x_*Sb_1−*x*_/Al_0.1_In_0.9_Sb QWs demonstrated a clear evolution from a characteristic two-dimensional electron gas to a behavior indicative of spatially separated carriers for x > 0.5, which they attributed to the onset of Type-II band alignment. This aligns perfectly with our band offset calculations, which show a valence band crossover ($∆E_{V}<0$) in this compositional range. Furthermore, our prediction of efficient tunable emission in the 3–4 µm range for Type-I structures is corroborated by the work of Nash et al. [3], who demonstrated room-temperature electroluminescence at 3.4 µm from an InSb/AlInSb QW light-emitting diode system corresponding to the (*x* = 1, *y* = 1) point in our design space. While direct experimental reports of luminescence across the whole compositional range we mapped are scarce, the existing data on specific points validate the overall trends predicted by our model. The close correspondence between our theoretical phase diagram and these experimental QW results underscores the predictive power of our approach. While all calculations are performed at low temperature to reflect intrinsic material parameters, the predicted emission wavelength will shift to longer wavelengths at elevated temperatures due to bandgap shrinkage (Varshni effect). Furthermore, increased carrier leakage from the quantum well and enhanced non-radiative recombination via phonon scattering will reduce internal quantum efficiency. However, the relative trends in tunability and Type-I/II behavior presented here remain qualitatively valid for room temperature operation. It provides a reliable, comprehensive roadmap for the molecular beam epitaxy (MBE) growth of next-generation, high-efficiency InAsSb/AlInSb optoelectronic devices. The sensitivity of III–V heterostructures to strain and temperature is well-documented, not only in antimonides but also in other ternary and quaternary systems [27]. The importance of strain and thermal management in optoelectronic devices has grown on mismatched substrates, where residual strain from thermal expansion mismatch can lead to unintended shifts in electronic structure and degradation in device performance. Translating the proposed lattice-matched designs into high-quality epitaxial structures requires addressing several material-specific challenges inherent to molecular beam epitaxy (MBE) of antimonide-based heterostructures. Precise flux control is paramount, particularly for the group-V elements As and Sb. Sb has a high surface mobility and a strong tendency to segregate and surface accumulate, which can lead to composition grading, interfacial broadening, and difficulty in achieving sharp heterointerfaces [28]. The growth of Al-c12ontaining barrier (AlInSb) introduces additional complexity due to Al’s high reactivity with residual oxygen and carbon, potentially forming non-radiative recombination centers that degrade luminescent efficiency [29]. Optimal substrate temperatures for InAsSb and AlInSb layers differ, necessitating careful temperature control to prevent interfacial intermixing and ensure crystal quality. Strain management is crucial; minor deviations from lattice matching can cause misfit dislocations and relaxation, degrading emission [30]. Experimental validation uses high-resolution X-ray diffraction (HR-XRD) to assess lattice constants, matching, and strain. Temperature-dependent photoluminescence (PL) spectroscopy provides key optoelectronic validation by directly measuring emission wavelength and linewidth, indicating interface quality and uniformity.

## 4. Conclusions

We have established a design of the lattice-matched InAs_1−*x*_Sb*_x_*/AlIn*_y_*Sb_1−*y*_ Type-I QWs that enables tunable emission across the 2–5 µm range via precise control of Sb (*x*) and In (*y*) contents. Strain energy minimization and band offset calculations indicate that Type-I alignment is achievable for *x* ≤ 0.40, whereas Type-II alignment dominates at higher Sb concentrations. This theoretical prediction, supported by excitonic transition calculations, provides a design toolkit for infrared optoelectronic devices. Future work will focus on validating MBE growth and characterizing devices to assess nonradiative losses at room temperature.

## Figures and Tables

**Table 1:** Parameters of binary III-V compounds. Lattice constant a: *E*_g_ is the band gap, *m*_e_ is the electron effective mass, *m*_hh_ is the heavy hole effective mass, *C*_11_ and *C*_12_ are the elastic stiffness constants, *a*_d_ and *b*_d_ are the deformation potentials, *ε* is the dielectric constant, *γ*_1_ and *γ*_2_ are the Luttinger parameters, *χ* the electron affinity, and the bowing parameters are *b*_AlInSb_ and *b*_InAsSb_.

Parameter	AlSb	InAs	InSb
*a* [Å]	6.1355	6.0583	6.4793
*E*_g_ [LT][eV]	2.386	0.420	0.240
*m*_e_/*m*_0_	0.14	0.024	0.013
*m*_hh_/*m*_0_	0.9	0.36	0.38
*C*_11_ [GPa]	87.69	83.29	66.08
*C*_12_ [GPa]	43.41	45.26	35.31
*a*_d_ [eV]	-	−5.08	−6.94
*b*_d_ [eV]	-	−1.8	−2
*ε*	11.21	14.3	17.2
*γ*_1_	4.15	20.4	36.3
*γ*_2_	1.01	8.3	16.1
*χ* [eV]	3.65	5.06	4.72
*b*_AlInSb_ [eV]	0.43	-	-
*b*_InAsSb_ [eV]	0.60	-	-

Parameter taken from [14].

## Data Availability

The original contributions presented in this study are included in the article. For further inquiries, please do not hesitate to contact the corresponding author.
